# Editorial: The Contribution of Postural Adjustments to Body Balance and Motor Performance

**DOI:** 10.3389/fnhum.2018.00487

**Published:** 2018-12-05

**Authors:** Eric Yiou, Alain Hamaoui, Gilles Allali

**Affiliations:** ^1^CIAMS, Univ. Paris Sud, Université Paris-Saclay, Orsay, France; ^2^CIAMS, Université d’Orléans, Orléans, France; ^3^Department of Neurology, Geneva University Hospitals and University of Geneva, Geneva, Switzerland; ^4^Division of Cognitive & Motor Aging, Department of Neurology, Albert Einstein College of Medicine, Yeshiva University, New York, NY, United States

**Keywords:** postural adjustments, body balance, Motor performance, motor control, human

## Introduction

The control of balance is crucial to maintain our posture and perform efficiently our daily motor tasks. Humans, as all terrestrial species, indeed evolve in a gravity field that permanently tends to induce postural destabilization by its attracting effect toward the center of the earth. Other external forces, arising from the environment such as those elicited by the acceleration of a train in which one stands, may also challenge body balance. In addition, many studies from the last decades and to date pointed out the disturbing effect of internal forces induced by voluntary segmental movements, even the tiniest ones such as single index movements. To keep the body (or part of it) steady, these disturbing forces must be compensated, at least partly. It is known that this compensation involves dynamical phenomena sub-served by highly-coordinated patterns of muscle activation/deactivation disseminated throughout the whole-body and called “postural adjustments.”

This research topic provides an up-to-date picture of the relationship between postural adjustments, body balance and motor performance in healthy (young and older adults) and pathological participants. It includes 35 contributions (28 original articles, 4 reviews, and 3 methods articles) which are separated into four sections: (1). Postural maintenance and multisensory integration, (2). Anticipatory postural adjustments associated with voluntary movement, (3). Postural adjustments associated with predictable and unpredictable external perturbation, (4). Gait assessment and rehabilitation in aging.

## Contributions

### Postural Maintenance and Multisensory Integration

This first section includes original articles investigating (i) the multisensory control of postural maintenance and movement, and (ii) how postural constraints influence sensory transmission, brain function and behavior. This section also reports articles focusing on new methods for measuring postural control.

The role of somato-sensorial information on postural maintenance and movement was investigated in the three following articles. In Teasdale et al. participants were exposed to vibrations of the Achilles tendons (sensing a backward body displacement) or *tibialis anterior* muscle tendons (sensing a backward body displacement), and pointed to a target (without vision). Main result showed that the target was undershot in both conditions. To explain these findings, authors proposed that ankles proprioceptive inputs are integrated into the upper limb planning, and that motor planning would then be adjusted on the basis of the expected consequences of movement on postural stability. Dupuy et al. investigated the perception of verticality and postural stability in patients with Ehlers-Danlos Syndrome, hypemobility type (EDS-HT). EDS-HT is a condition characterized by generalized joint hypermobility, variable skin hyperextensibility, and impaired proprioception. Authors showed that EDS-HT was associated with changes in the relative contributions of somatosensory and vestibular inputs to verticality perception. Moreover, postural impairment observed in these patients was offset, at least partially, by wearing somatosensory orthoses (i.e., compressive garments and insoles). Maitre and Paillard investigated the effects of age and physical activity status on balance control adaptation to different supporting surfaces, thus modifying sensory inputs from the feet. Elderly were more disturbed than young adults when standing on a foam surface, probably because of the structural and functional involutions of plantar cutaneous sole and foot with aging. In contrast, no difference between active and sedentary groups was found, probably because the postural task was not difficult enough to discriminate these two groups. Now, it is possible that alternative data analysis methods may be necessary to reveal between-group differences during standing. This point is stressed in the three following articles.

Hansen et al. compared various entropy measures to assess the dynamics and complexity of center-of-pressure (COP) displacements during standing. These entropy measures describing COP regularity at different scales were compared to traditional measures of COP variability. Results showed that entropy measures analysis techniques are more sensitive in the incremented time-series compared to classical parameters and entropy measures of original time-series. Such surplus value of the non-linear dynamical analysis of COP trajectories to gain insight into the mechanisms involved in the control of bipedal posture was further stressed by Isableu et al. Earlier findings based on COP classical variables showed that gymnasts exhibited a better control of balance but only in demanding stances. These authors showed that the effect of expertise in Gymnastic could be uncovered in non-demanding postural stance, from the analysis of non-linear dynamic patterns of COP trajectories. In addition, dos Anjos et al. tested whether the distribution of ankle muscle activity differs between aged and young subjects during standing. Surface EMGs were sampled at multiple skin locations from *tibialis anterior, soleus* and medial, and lateral *gastrocnemius* muscles in young adults and elderly. Their results showed that the duration and size of active muscle volume, as quantified from spatial distribution of surface EMGs, discriminate well aged from young individuals. Their results further suggest that different conclusions on active control of standing posture may be drawn depending on skin location from where EMGs are collected.

The influence of vision on postural stability was investigated in the two following studies. Chatard et al. investigated the impact of unilateral vs. bilateral age-related macular degeneration (AMD) on postural sway, and the influence of different visual conditions. Main result showed that bilateral and unilateral AMD subjects had lower stability than healthy elderly, a finding that led authors to suggest that ADM subjects may have poor postural adaptive mechanisms. Olivier et al. evaluated the relative contribution of visual information to horseback riders' postural stability and the coordination modes to regulate balance according to expertise. Results showed that professional riders exhibited greater overall postural stability than Club riders. In particular, head variability was lower in Professional in visually altered conditions, suggesting a greater ability to use vestibular and somesthetic information according to task constraints with expertise. Professional exhibited specific coordination modes that, unlike the Club riders, departed from pure in-phase and anti-phase patterns and depended on visual conditions. These findings provide evidence of major differences in the sensorimotor processes contributing to postural control with expertise in horseback riding.

Thus, as shown in the above-reported articles, sensory signals from visual, vestibular, and mechano-receptors are crucial to control posture and balance. In turn, postural constraints are crucial for gating sensory information to the brain, as shown in the two following articles. Mouchnino et al. investigated the effect of plantar sole unweighting on the sensory transmission to the cortical areas during standing. Cortical somatosensory evoked potentials were recorded following cutaneous plantar sole stimulation in different conditions of unweighting (100% bodyweight BW to 40% BW). Contrary to what was expected, authors found an attenuation of sensory information when the BW was unweighted to 41%. Authors argued that this attenuation was not solely due to the absence of forces acting on the sole of the feet but rather to the current relevance of the afferent signals related to balance constraints. The effect of posture on brain activity was also addressed in the review of Thibault and Raz. These authors challenged the tacit assumption that brain processes and cognitive performance are comparable across a spectrum of posture. An integrative synthesis regarding the increasingly prominent influence of imaging postures on autonomic function, mental capacity, sensory thresholds, and neural activity, is provided in the review. Arguing that neuroimagers and cognitive scientists could benefit from considering the influence posture wields on both general functioning and brain activity, authors examined existing imaging technologies and the potential of portable and versatile imaging devices. Finally, authors discussed ways that accounting for posture may help unveil the complex brain processes of everyday cognition.

### APAs Associated With Voluntary Movement

This second section reports original articles and reviews focusing on the postural phenomena occurring before movement onset (the so-called “anticipatory postural adjustments,” APAs). These phenomena are known to be crucial for stability maintenance and motor performance. A better knowledge of their characteristics is therefore of uttermost importance to the researchers and the clinicians. The articles of this section include various experimental models for the investigation of APAs (from whole-body movements to distal movements, discrete, or cyclical), as well as a large panel of populations (healthy or pathological). This section thus offers the reader a broadened view on the different facets of APAs.

Gait initiation (GI) is an experimental model that is classically used to investigate postural control during a whole-body movement. It has received a large consideration in this topic. GI is a functional task, which corresponds to the transient phase between static posture and steady-state locomotion. It includes COP shift toward the swing foot (frontal plane shift) and the heels (sagittal plane shift), which allows establishing stable body progression and desired step length/velocity. Honeine et al. investigated the neuro-mechanical process that underlies APAs in the frontal plane. These authors demonstrated that the coordinated activation of hip abductors and ankle dorsiflexors was responsible for the anticipatory COP shift toward the swing leg, and for setting the contact position for the swing foot. These findings corroborate with Bancroft and Day, which proposed a “throw-and-catch” hypothesis of human gait where APAs are organized to generate momentum for the body to fall ballistically along a specific trajectory during the step. The hypothesis predicted a strong coupling between APAs and step location. It was validated by the results of their experiments where participants had to step as accurately as possible onto targets that required either different step directions or different step lengths, and that could jump to a new location during APAs.

In addition to step location, APAs are also sensitive to the potential instability elicited by an environmental constraint in the form of an obstacle to clear. This is a main result of Yiou et al.'s study which proposed a mechanical model of the body to highlight the role of mediolateral APAs in stability control during GI over obstacles of different height and distance. Results showed that postural stability at the time of foot-contact was the same in all conditions of obstacle height and distance, which was made possible thanks to adapted modulation of mediolateral APAs. This finding led authors to suggest that the CNS is able to predict the potential instability elicited by obstacle features and that it scales APAs parameters accordingly. This idea that the CNS is able to modulate APAs so as to maintain an equivalent stability was further reinforced in Caderby et al. which focused on the effects of changing initial body weight distribution between legs (with external loads) on mediolateral stability during GI.

Besides such external constraints, APAs and related-postural stability and motor performance also depend on postural chain mobility. This is the main result of the four following studies, which experimentally manipulated the mobility of various articulations of the postural chain.

Delafontaine et al. reported that APAs associated with GI decreased when the stance ankle mobility was reduced with an ankle foot orthosis (AFO). As a consequence, the vertical braking of the COM during swing phase (an indicator of postural control) and motor performance were both affected. In contrast, the mediolateral stability was increased thanks to a larger step. Authors proposed that rehabilitation perspective could be to prescribe passively- or actively-powered assistive AFO. The results obtained by Hamaoui and Alamini-Rodrigues corroborate with these findings. These authors investigated the influence of cervical spine mobility (CSM), which was modulated by means of splints, on the focal and postural components of the sit-to-stand transition. These authors showed that restricted CSM leaded to a lower motor performance and a reorganization of the APAs. Authors proposed that preserving the articular free play of the cervical spine might be useful to favor sit-to-stand performance and autonomy. In a complementary study, the same authors (Hamaoui and Alamni-Rodrigues) focused on the influence of a restricted mobility of the trunk on the sit-to-stand organization. This time, restricted mobility was induced by means of varying the muscular tension along the trunk. Results showed that beyond a given level, higher trunk muscular tension resulted in longer APAs. In contrast, motor performance remained unchanged. Restricted trunk mobility thus requires an adaptation of APAs programming to keep the same level of performance. These findings may have implications in treatment strategies aimed at preserving functional autonomy in pathologies including a rise of muscular tension.

Ditcharles et al. also investigated the relationship between postural chain mobility and postural control in a dynamical task but, this time, mobility was experimentally sought to be increased by a manual thoracic spine manipulation (High Velocity Low Amplitude, HVLA). These authors showed that, despite the HVLA-manipulation was efficient to improve spine mobility, APAs and speed performance associated with GI were reduced. A neural effect induced by the manipulation, possibly mediated by a transient alteration in the early sensory-motor integration, might have masked the potential mechanical benefits associated with increased spine mobility.

Balance control is known to be impaired in elderly (and particularly in fallers) and patients with Parkinson's disease (PD). The results of the following studies contribute to a better understanding of GI impairments in these populations. New approaches were also proposed to improve dynamic balance in elderly.

As stressed by Cohen et al. freezing of gait in PD has been linked with deficits in inhibitory control, but causal mechanisms are not established. Freezing at GI (start hesitation) is often accompanied by multiple APAs. Cohen et al. investigated how inhibition deficits could contribute to freezing in PD patients. Their findings revealed that start hesitation is not caused by multiple APAs *per se*, but may be associated with difficulty recovering from multiple APAs, due to difficulty releasing a previously inhibited step. Tisserand et al. highlighted changes in the capacity of elderly fallers vs. non-faller to step quickly under a choice stepping reaction-time test (CSRT). Authors found that fallers used a more cautious APAs strategy than non-fallers, which resulted in longer step duration and larger margin of stability at foot-off. Such a choice in balance strategy probably comes from muscular limitations and/or a higher fear of falling and paradoxically indicates an increased risk of fall.

Kaminski et al. also stressed the need to evaluate older adults' ability to maintain balance and examining new approaches to counteract age-related decline in balance control for fall prevention and healthy aging. These authors investigated the influence of transcranial direct current stimulation applied over the leg area of the primary motor cortex (M1) on dynamic balance task (DBT) learning of healthy elderly. Results indicated that this method did not elicit DBT learning enhancement. However, a regression analysis revealed that DBT performance can be predicted by the kinematic profile of the movement, a finding that may provide new insights for individualized approaches of treating balance and gait disorders. Finally, Lelard et al. showed that leg electromyographic and COP path pattern during GI could discriminate yoga practitioners among physically active older subjects.

The above papers investigated the characteristics of APAs associated with complex movements that induced substantial perturbation of whole-body balance. Such APAs involve the coordination of muscles that may be disseminated through the whole-body, and were thus called *inter-limb* APAs. In their review, Cavallari et al. questioned the role of APAs associated with movements involving tiny mass limbs that do not endanger whole-body balance (e.g., index finger extension etc.). Such APAs involve muscles acting on the proximal joints and correspond to *intra-limb* APAs. These authors proposed that these APAs would be involved in refining movement precision by granting a proper fixation of body segments proximal to the moving one. In line with this idea, Bruttini et al. proposed that the higher precision in pointing with the preferred vs. non-preferred hand would be due to an earlier intra-limb APA in the preferred arm. Cavallari et al. further argued that inter- and intra-limb APAs are manifestations of the same phenomenon, and that APAs and prime mover activation are driven by a shared motor command.

Finally, it is noteworthy that, in all the above-reported articles, APAs features associated with *discrete* movements were performed. However, in daily life, *rhythmical* voluntary movements involving distant limbs may also be performed. Voluntary movement may also be combined with postural tasks requiring more or less attentional resources. The two following articles focused on how the brain organizes movement production under the neural/attentional constraints inherent to such combined tasks. For example, rhythmical isodirectional flexion-extensions of ipsilateral hand and foot are known to be easily performed, while antidirectional movements require attentive effort and irresistibly tend to reverse into isodirectional when frequency increases. In their review, Baldissera and Tesio reported results of experiments indicating that this direction-dependent easy-difficult dichotomy is caused by interference of APAs commands associated to movements of one limb with voluntary commands to the other limb. Huang et al. investigated how brain networks are organized to optimize a suprapostural motor task when the postural load increases and shifts postural control into a less automatic process. A force-matching task was made from a level surface (a relative automatic postural task) and from a stabilometer board while maintaining balance at a target angle (a relatively controlled postural task). Increasing stance difficulty altered the neurocognitive processes in executing postural-suprapostural task. Suprapostural performance was not degraded by increase in postural load, due to (1) increased effectiveness of information transfer, (2) an anterior shift of processing resources toward frontal executive function, and (3) cortical dissociation of control hubs in the parietal-occipital cortex for neural economy.

### Postural Adjustments Associated With Predictable and Unpredictable External Perturbation

This third section reports original articles and a review that focus on the adaptability of the postural response associated with an external perturbation applied under various sensorimotor contexts.

Abboud et al. proposed a systematic review to assess the quality of evidence of studies investigating trunk neuromuscular responses to unexpected trunk perturbation applied under condition of muscle fatigue, spinal tissue creep or in the presence of low back pain. Authors stressed that if the literature provides some evidence with regard to trunk adaptions in a context of spinal instability, most of the evidence was inconclusive due to high methodological between-studies heterogeneity. The same research team (Abboud et al.) further investigated, in an original article, the influence of lumbar muscle fatigue on trunk adaptations to repeated sudden external perturbations. By using high-density electromyography technique, these authors showed that muscle fatigue leads to reduced spatial distribution of back muscle activity and suggest a limited ability to use across-trial redundancy to adapt EMG reflex peak and optimize spinal stabilization using retroactive control.

In Camernik et al. immediate and transitionary effects of supportive hand contacts during continuous perturbations of stance were elicited by automated waist-pulls. Kinematical analysis showed that the utilization of the supportive hand contacts facilitates balance control, and that postural readjustments after the release of the handle occurred at different time scales in the hip, knee, and ankle joints. These findings corroborate with the study of Sozzi et al. where continuous perturbations were elicited by sinusoidal platform displacement cycles administrated to eyes-closed and eyes-opened participants. Authors showed that ankle muscles activity decreased over time, and that the level of attenuation depended on the muscle (*soleus* or *tibialis anterior*) and on the nature of the response (reflex or anticipatory). Vision more than doubled speed and amount of EMG adaptation, rapidly enhanced body segment coordination, and crucially reduced head displacement.

Globally, these findings bring new and important insights on the mode of amplitude- and time-modulation of motor output during adaptation in a balancing task, advocate for new protocols for assessing flexibility of balance strategies, and provide references for addressing balance problems in patients with movement disorders.

### Gait Assessment and Rehabilitation in Aging

Gait disorders are a major source of disability in aging affecting more than 30% of non-demented older adults. Neurological (i.e., Parkinson's disease) and non-neurological causes contribute to gait disorders in aging (Verghese et al., 2006). Osteoarthritis is a major cause of non-neurological gait disorders (Snijders et al., 2007). Barden et al. compared gait parameters between older adults with and without bilateral knees osteoarthritis. They demonstrated a loss of gait regularity in older adults with bilateral knees osteoarthritis in comparison to healthy older adults. This study represents a good example on how gait measurement has improved our understanding of the mechanisms of gait disorders in aging. However, there is a need for guidelines on gait measurement in aging and for studies assessing the reliability of gait parameters. In this research topic, an international panel of experts proposes a consensus guideline for gait evaluation and spatiotemporal gait analysis, as well as reference values for healthy older adults (Beauchet et al.). In a methodological study, Szturm et al. assessed the reliability of spatiotemporal gait parameters recorded on a treadmill, while using an original dual task paradigm (i.e., walking while performing a cognitive task) relying on visuomotor and visuospatial tasks.

Various rehabilitative pharmacological and non-pharmacological interventions have been developed in older adults with and without cognitive impairment to tackle gait disorders and falls (Allali and Verghese, 2017). This research topic includes a promising non-pharmacological rehabilitative method based on the use of visual kinematic perturbations (Luu et al.). The study findings suggest an interesting adaptation learning that needs further investigations in disease population.

## Conclusion

Beside their basic interest of unveiling the mechanisms behind motor control, results from the investigations of this topic are relevant to develop new methods or tools to improve postural stability and motor performance, with applications in the fields of neurodegenerative conditions, rehabilitation, ergonomics, and sports sciences. Establishing the interaction between postural control, locomotion, and cognition has important clinical implication, especially in term of falls prevention, and will improve our knowledge on the underlying neural correlates.

## Author Contributions

All authors listed have made a substantial, direct and intellectual contribution to the work, and approved it for publication.

### Conflict of Interest Statement

The authors declare that the research was conducted in the absence of any commercial or financial relationships that could be construed as a potential conflict of interest.
